# Tumor cure by radiation therapy and checkpoint inhibitors depends on pre-existing immunity

**DOI:** 10.1038/s41598-018-25482-w

**Published:** 2018-05-03

**Authors:** Marka R. Crittenden, Lauren Zebertavage, Gwen Kramer, Shelly Bambina, David Friedman, Victoria Troesch, Tiffany Blair, Jason R. Baird, Alejandro Alice, Michael J. Gough

**Affiliations:** 1 Earle A. Chiles Research Institute, Providence Portland Medical Center, Portland, OR 97213 USA; 2 The Oregon Clinic, Portland, OR 97213 USA; 3 Molecular Microbiology & Immunology, Oregon Health & Science University, Portland, OR 97239 USA

## Abstract

Radiation therapy is a source of tumor antigen release that has the potential to serve as an endogenous tumor vaccination event. In preclinical models radiation therapy synergizes with checkpoint inhibitors to cure tumors via CD8 T cell responses. To evaluate the immune response initiated by radiation therapy, we used a range of approaches to block the pre-existing immune response artifact initiated by tumor implantation. We demonstrate that blocking immune responses at tumor implantation blocks development of a tumor-resident antigen specific T cell population and prevents tumor cure by radiation therapy combined with checkpoint immunotherapy. These data demonstrate that this treatment combination relies on a pre-existing immune response to cure tumors, and may not be a solution for patients without pre-existing immunity.

## Introduction

Cancer cell lines grown *in vitro* and implanted into genetically identical cohorts of mice provides a controlled system to establish matched tumors for preclinical modeling. However, the process of tumor implantation has long been known to generate an anti-tumor immune response that subsequently impacts tumor growth and development. Bursuker and North demonstrated that initial effector CD8 responses to tumor implantation could be transferred to other hosts to control matched tumors, and these initial anti-tumor CD8 T cell responses were later accompanied by CD4 regulatory T cell responses that suppressed the CD8 effectors^1–3^. If the initial immune response to tumor implantation is sufficiently strong, tumors spontaneously regress in immune competent animals, but remain capable of progressive growth in immune deficient animals^4,5^. Where tumors grow despite strong immunogenicity, immune control of the tumor can be reinvigorated by interventions such as radiation therapy^6,7^, which generates large-scale release of tumor antigens following cancer cell death. The full response to radiation therapy in murine models is dependent on CD8 T cell immunity, and radiation therapy synergizes well with T cell targeted immunotherapies for control of local and distant disease^8,9^.

To investigate the immune responses activated following radiation therapy of established tumors, we tested approaches to eliminate the immune response to tumor implantation so radiation could be studied in isolation. We found that by eliminating pre-existing immune responses generated by tumor implantation, the combination of radiation therapy plus checkpoint inhibition was no longer effective. Tumor implantation resulted in a tumor-resident population of antigen-specific T cells that correlated with response to treatment. These data have significant implications for current attempts to use radiation therapy plus checkpoint inhibitors to generate immune-mediated tumor cures in patients lacking pre-existing immunity.

## Results

**Figure 1:**
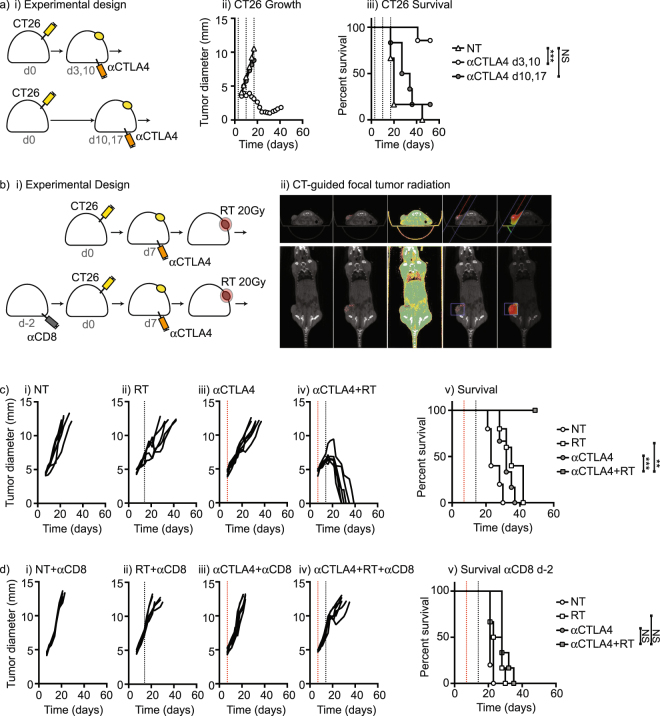
The addition of radiation therapy permits control of tumors after checkpoint failure that is dependent on CD8 T cells at tumor implantation. (**a**) BALB/c mice were implanted with CT26 tumors and left untreated (NT) or treated with anti-CTLA4 on d3 and d7, or on d7 and d14. Graphs show i) Average tumor size and ii) survival. (**b**) i) BALB/c mice were randomized to receive CD8 depleting antibodies 2 days prior to implantation with CT26 tumors, and groups randomized to receive anti-CTLA4 on d7 and radiation therapy on d14. ii) Radiation therapy was delivered using CT guidance to deliver a focal therapeutic dose of 20 Gy with minimize dose to radiosensitive structures. Graphs show growth and survival of individual tumors in (**c**) control treated and (**d**) anti-CD8 treated mice. Key: *p < 0.05; **p < 0.01; ***p < 0.001; ****p < 0.0001; NS = not significant.

Mouse tumor models are known to vary in their immunogenicity and their responsiveness to immunotherapies; however, even responsive tumors can become unresponsive as tumors progress. Mice bearing CT26 colorectal carcinomas were responsive to single agent anti-CTLA4 treatment initiated closely following tumor implantation (p < 0.01) (Fig. 1a). However, delaying treatment with anti-CTLA4 removed all efficacy of this treatment. This is consistent with a decay of the immune response initiated following tumor implantation. Alternatively, failure of delayed treatment could also reflect accumulating tumor burden or an acquired suppressive tumor environment. Radiation therapy has been proposed as a potent partner for immunotherapy in part due to release of tumor antigens following radiation-induced cancer cell death. The addition of CT-guided radiation therapy to anti-CTLA4 treatment according to the optimum schedule^10^ (Fig. 1b) resulted in cure of tumor-bearing mice while each therapy was ineffective alone (Fig. 1c). Since anti-CTLA4 in murine models predominantly functions to deplete T regulatory cells^11,12^ and does not expand new T cell responses^10^, we hypothesized that in this setting radiation therapy acted to re-expand T cells generated by tumor implantation, rather than generating new anti-tumor T cell responses. To test this hypothesis, in a comparison group we depleted CD8 T cells prior tumor implantation, rather than the standard approach of T cell depletion prior to RT, and treated with anti-CTLA4 and RT (Fig. 1b). Interestingly, in mice depleted of CD8 T cells prior to tumor implantation the combination of anti-CTLA4 and RT was ineffective (Fig. 1d). These data suggest that the synergy between radiation therapy and checkpoint inhibitor therapy was dependent on pre-existing immunity generated at tumor implantation.

**Figure 2:**
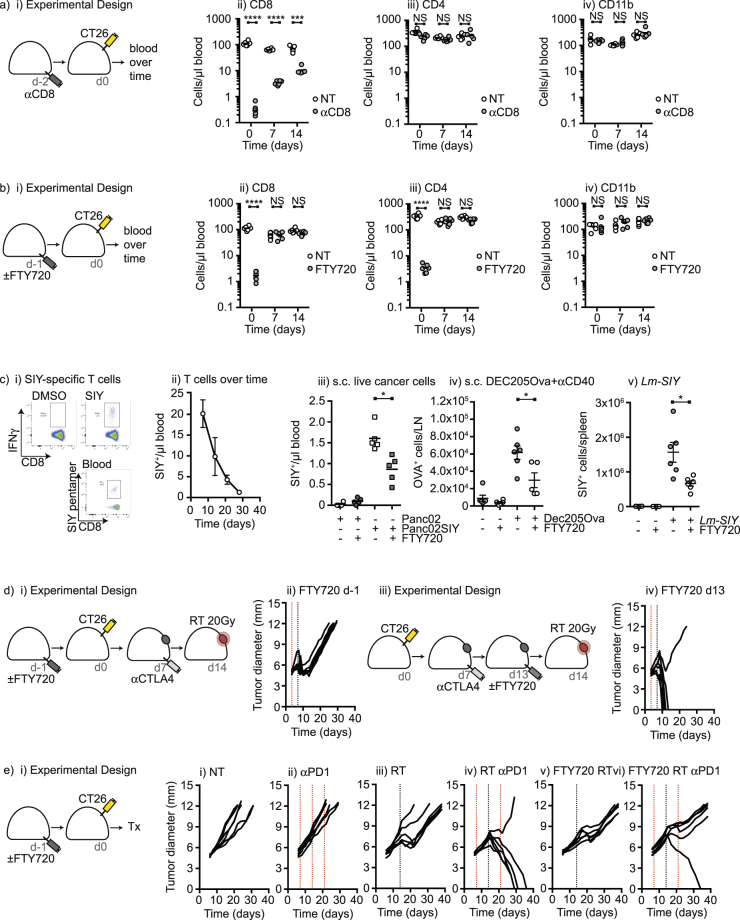
FTY-720 immunosuppression at tumor implantation eliminates tumor control by subsequent radiation therapy and checkpoint inhibition. (**a**) BALB/c mice were randomized to receive CD8 depleting antibodies 2 days prior to implantation with CT26 tumors. Graphs show quantitative analysis of i) CD8^+^, ii) CD4^+^ and iii) CD11b^+^ cells over time following CD8 depletion. (**b**) i) BALB/c mice were randomized to receive FTY-720 1day prior to implantation with CT26 tumors. Graphs show quantitative analysis of ii) CD8^+^, iii) CD4^+^ and iv) CD11b^+^ cells over time following FTY-720 administration. (**c**) i) C57BL/6 mice were implanted with Panc02-SIY tumors and analyzed for SIY-specific T cells in the spleen by ICS for IFNγ, or in the blood using an SIY-pentamer. ii) Time course of the SIY-specific response in the peripheral blood of mice implanted with Panc02-SIY through tumor growth. Mice were randomized to treatment with FTY-720 or left untreated, then vaccinated 1day later with iii) subcutaneous injection of Panc02-SIY, iv) subcutaneous injection of Dec205-Ova + anti-CD40, or v) intravenous administration of Lm-SIY. Graphs show antigen-specific cells in blood, LN or spleen, respectively. d) i) Mice were implanted with CT26 and treated with anti-CTLA4 on d7 and radiation on day 14. Groups were randomized to treatment with i-ii) FTY-720 1day prior to tumor implantation or iii-iv) 1day prior to radiation. (**e**) Mice were implanted with CT26 and i) left untreated; ii) treated with anti-PD1 on d7, 14 and 21; iii) treated with radiation on day 14; iv) treated with the combination; iv) treated with FTY720 1day prior to implantation and radiation on d14. v) treated with FTY720 1day prior to implantation, anti-PD1 on d7, 14 and 21 and radiation on d14. Key: *p < 0.05; **p < 0.01; ***p < 0.001; ****p < 0.0001; NS = not significant. Each symbol represents one animal.

When monitoring the recovery of CD8 T cells following antibody-mediated depletion we noted that CD8 T cell numbers had not fully recovered and remained significantly lower than control mice at the time of immunotherapy and radiation therapy (p < 0.005) (Fig. 2a), potentially explaining the poor efficacy of the combination in these mice. As an alternative approach to block the immunogenicity of tumor implantation, we made use of the immunosuppressive drug FTY-720. This drug blocks S1P1-mediated exit of lymphocytes from lymph nodes, and also blocks drainage of lymphocytes from peripheral sites via lymphatic endothelia. A single treatment with FTY-720 resulted in two log fold selective loss of lymphocytes from the peripheral blood 1 day later (p < 0.001), and full recovery by 7 days following treatment (Fig. 2b). To test the consequence of FTY-720 treatment on the immune response to tumor implantation, we used Panc02 cells engineered to express the model antigen SIY. Following implantation of these tumors there is a readily detectable SIY-specific CD8 T cell response in the spleen and peripheral blood without any additional treatment (Fig. 2ci), and the number of these antigen-specific cells in circulation declines in number as the tumor progresses (Fig. 2cii). This T cell response to tumor implantation was not unique to the Panc02-SIY model, as it also occurred in spontaneous pancreatic tumors derived from mice that express SIY in the pancreas, and in spontaneous pancreatic tumors engineered to express the model antigen SIINFEKL (Supplementary Figure 1). Administration of a single dose of FTY-720 24 hours prior to Panc02-SIY tumor implantation significantly decreased antigen-specific responses detectable in the peripheral blood (Fig. 2ciii) consistent with the effect of FTY-720 on subcutaneous vaccination with DEC-205-Ova plus anti-CD40 and on systemic vaccination with attenuated *L. monocytogenes* (Fig. 2civ-v). To investigate the effect of this inhibition on the response to treatment, groups of mice received FTY-720 1 day prior to tumor implantation or 1 day prior to radiation therapy (Fig. 2d). Treatment prior to tumor implantation entirely blocked the efficacy of anti-CTLA4 plus RT treatment (Fig. 2di-ii) while treatment with FTY-720 immediately prior to RT had no effect – tumors in these mice were effectively rejected by anti-CTLA4 plus RT (Logrank survival d-1 vs d13 FTY-720 p < 0.05) (Fig. 2diii-iv). These data are consistent with our data with CD8 depletion (Fig. 1), suggesting T cells generated at implantation are critical to efficacy. To determine whether this mechanism was specific to anti-CTLA4 or shared with other checkpoint inhibitors, mice were treated with anti-PD1, RT or the combination, which resulted in tumor cure only in mice treated with the combination (Fig. 2e). As with the combination of RT and anti-CTLA4, treatment with FTY-720 prior to tumor implantation prevented tumor cure by the combination of RT with anti-PD1 (Logrank survival RT vs RT + anti-PD1 p < 0.05; RT vs RT + anti-PD1 + FTY-720 Not significant) (Fig. 2e). These data demonstrate that immunosuppression at the time of tumor implantation decreased the antigen-specific response initiated as an artifact of tumor implantation, and prevented tumor control by radiation therapy plus checkpoint inhibitors.

**Figure 3:**
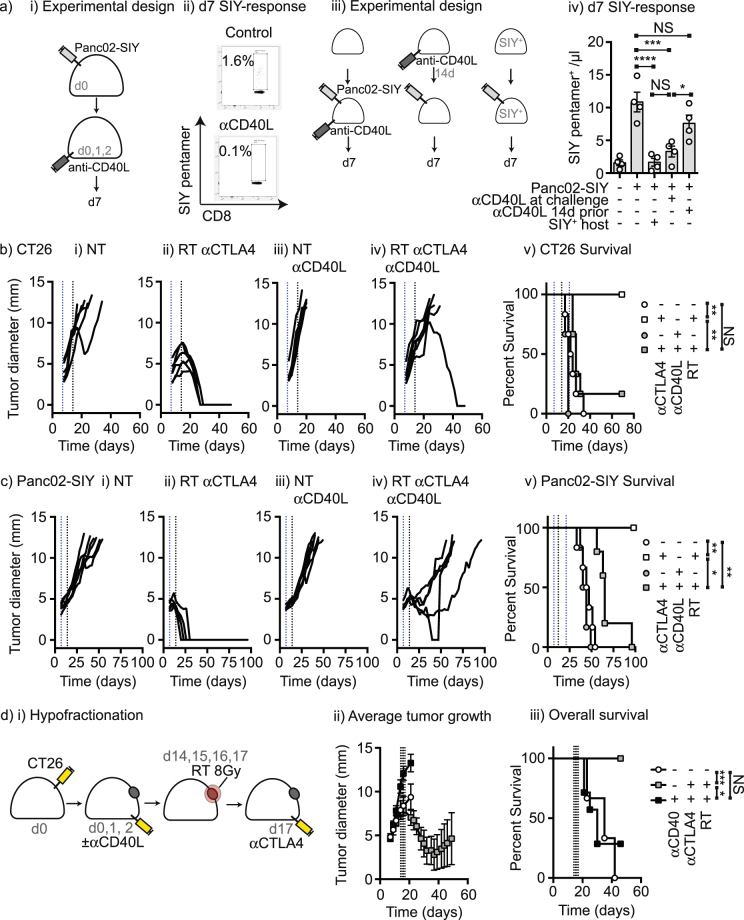
Anti-CD40L at tumor implantation eliminates tumor control by subsequent radiation therapy and checkpoint inhibition. (**a**) (i) Mice were implanted with Panc02-SIY tumors on d0 and left untreated or treated with 100 µg anti-CD40L on d0, d1 and d2. ii) SIY-specific pentamer binding cells in the peripheral blood 7d following tumor implantation in control and anti-CD40L-treated mice. iii) Panc02-SIY were implanted into wild-type mice on d0 and mice were left untreated or treated with 100 µg anti-CD40L on d0, 1, and 2 or two weeks previously (on d-14, -13, -12). Control mice received no tumor implantation, or Panc02-SIY implantation into SIY^+^ mice that were tolerant to SIY. iv) SIY-specific pentamer binding cells in the peripheral blood 7d following tumor implantation. (**b**) Mice were implanted with CT26 and i) left untreated; ii) treated with anti-CTLA4 on d7 and radiation on day 14; iii) treated with 100 µg anti-CD40L on d0, 1, and 2 iv) treated with 100 µg anti-CD40L on d0, 1, and 2 then treated with anti-CTLA4 on d7 and radiation on day 14; v) Overall survival of treatment groups. (**c**) Mice were implanted with Panc02-SIY and i) left untreated; ii) treated with anti-CTLA4 on d7 and radiation on day 14; iii) treated with 100 µg anti-CD40L on d0, 1, and 2 iv) treated with 100 µg anti-CD40L on d0, 1, and 2 then treated with anti-CTLA4 on d7 and radiation on day 14; v) Overall survival of treatment groups. (**d**) Mice were implanted with CT26 and left untreated, treated with 8 Gy radiation on d14, 15, 16 and 16, and anti-CTLA4 on d17, or treated with 100 µg anti-CD40L on d0, 1, and 2 then treated with 8 Gy radiation on d14, 15, 16 and 16, and anti-CTLA4 on d17. Graphs show ii) Average tumor growth and iii) Overall survival. Key: *p < 0.05; **p < 0.01; ***p < 0.001; ****p < 0.0001; NS = not significant.

While FTY-720 was effective at blocking the effect of therapy, it was not entirely able to block T cell responses (Fig. 2c), consistent with prior reports^13^. To develop a model of silent tumor implantation, we applied an approach widely used in the transplant literature. Treatment of mice with anti-CD40L permits allograft transplant by preventing T cell responses to the graft without depleting the antigen-specific cells^14–16^. Mice were implanted with live Panc02-SIY cancer cells and treated with anti-CD40L on d0, 1 and 2 (Fig. 3ai). Anti-CD40L treatment prevented the emergence of SIY-specific cells in the peripheral blood (Fig. 3aii). This was a transient immunosuppression, since mice remained capable of making antigen-specific immune responses if the tumor was implanted two weeks after anti-CD40L treatment (Fig. 3aiii-iv). The T cell response in mice treated with anti-CD40L at implantation appeared to be above background, but was not significantly different from that detected in SIY-tolerant mice (Fig. 3aiv), suggesting that this represents a relatively ‘silent’ tumor implantation. Mice given Panc02-SIY plus anti-CD40L then two weeks later rechallenged with *L. monocytogenes* expressing SIY generated a SIY-specific T cell response that was not significantly different from naïve mice, indicating that SIY-specific T cells had not been deleted (Supplementary Figure 2). However, in mice previously primed by implantation with Panc02-SIY without anti-CD40L the mice exhibited a boosted response (Supplementary Figure 2). These data suggest that anti-CD40L treatment blocks the initial T cell response to antigens without deleting antigen-specific cells, as previously reported in transplant models^17^. To determine whether this affected the result of combination therapy, mice were randomized to receive anti-CD40L blockade at tumor implantation or control treatment, and followed with combination therapy (Fig. 3b and c). Treatment with anti-CD40L at tumor implantation blocked the efficacy of radiation therapy combined with anti-CTLA4 in the CT26 tumor model (p < 0.01) (Fig. 3b) and the Panc02-SIY tumor model (p < 0.05) (Fig. 3c). While there is discussion in the literature, alternate hypofractionated dosing schemes have been proposed to be superior to single large doses to generate immune responses following radiation therapy^18^. To determine whether pre-existing immunity is necessary for tumor control by checkpoint inhibitors and radiation delivered in an alternative hypofractionated regimen, CT26 tumors were implanted in mice in the presence or absence of anti-CD40L then treated with 8 Gy radiation on 4 consecutive days and treated with anti-CTLA4 delivered in the concurrent timing that has proven superior in this scheme^19^. Tumor control by 8 Gy x4 plus anti-CTLA4 was also eliminated when pre-existing immunity was blocked (p < 0.05) (Fig. 3d), indicating that this treatment regimen is also dependent on pre-existing immunity for tumor control. These data using anti-CD40L to block the immune response at tumor implantation support our prior experiments with CD8 depletion and FTY-720 demonstrating that immune responses at implantation are critical to the efficacy of therapy. It remains to be determined whether anti-CD40L blockade at tumor implantation affects all immunotherapies in this class, or is limited to the cell lines and combinations treated here; however, we propose that experiment such as these are important to determine the ability of treatments to initiate new immune responses versus improve tumor control by pre-existing immunity.

**Figure 4:**
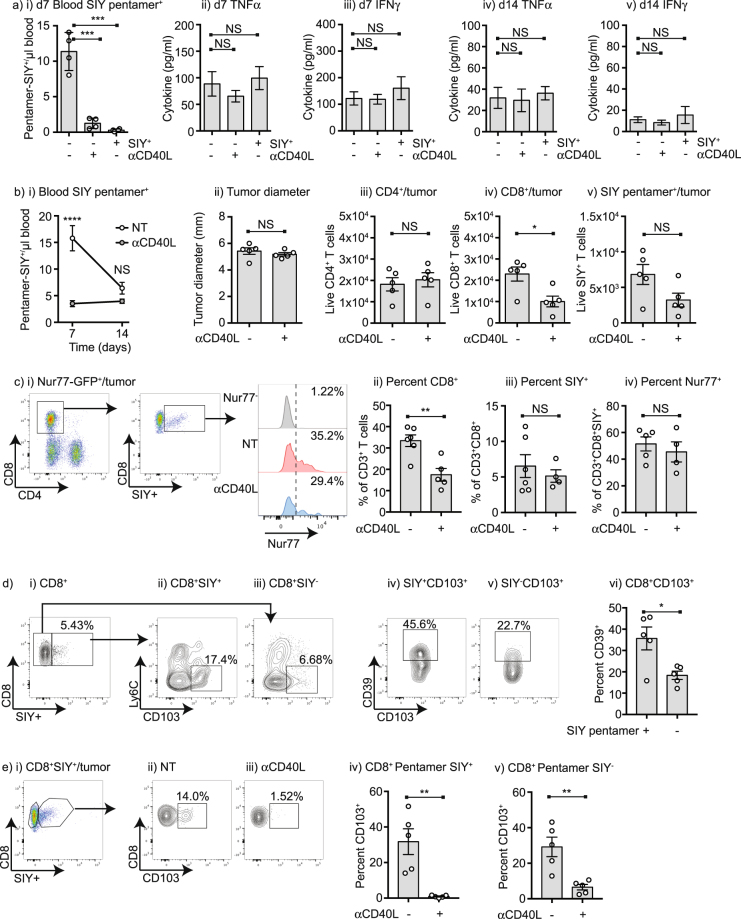
Anti-CD40L at tumor implantation prevents the development of Trm in the tumor environment. (**a**) i) C57BL/6 mice were implanted with Panc02-SIY tumors on d0 and left untreated; implanted with Panc02-SIY tumors and treated with 100 µg anti-CD40L on d0, d1 and d2; or Panc02-SIY tumors were implanted into SIY^+^ mice that were tolerant to SIY. Graph shows SIY-specific pentamer binding cells in the peripheral blood 7d following tumor implantation. Tumors were harvested on ii-iii) d7 or iv-v) d14 following implantation and analyzed for tumor cytokines by multiplex bead assay. Graphs show levels of TNFα or IFNγ in the tumors. (**b**) C57BL/6 mice were implanted with Panc02-SIY tumors on d0 and left untreated or treated with 100 µg anti-CD40L on d0, d1 and d2. i) Mice were analyzed for SIY-specific cells in the peripheral blood on d7 and d14 following tumor challenge. ii) Tumor size at d14 following challenge. d14 tumors were harvested and analyzed for the number of tumor infiltrating iii) CD4+ T cells, iv) CD8+ T cells, v) SIY-specific T cells. (**c**) C57BL/6 Nur77-GFP^−^ mice or Nur77-GFP^+^ mice were implanted with Panc02-SIY tumors on d0 and left untreated or treated with 100 µg anti-CD40L on d0, d1 and d2. i) CD8^+^SIY pentamer^+^ T cells were analyzed for Nur77-GFP expression. Graphs show the percent of ii) CD8^+^ T cells, iii) SIY^+^ T cells and iv) Nur77-GFP^+^ T cells. (**d**) C57BL/6 mice were implanted with Panc02-SIY tumors and i) tumor-infiltrating CD8^+^ T cells were gated into ii) SIY pentamer^+^ or iii) SIY pentamer^-^ cells, and analyzed for Ly6C and CD103 expression. CD39 expression in v) CD8^+^SIY pentamer^+^ CD103^+^ T cells, vi) CD8^+^SIY pentamer^−^ CD103^+^ T cells. vii) Summary of CD39^+^ T cells. (**e**) C57BL/6 mice were implanted with Panc02-SIY tumors on d0 and left untreated or treated with 100 µg anti-CD40L on d0, d1 and d2. i) Tumor-infiltrating CD8^+^SIY pentamer^+^ T cells were analyzed for CD103 expression in ii) untreated (NT) and iii) anti-CD40L treated animals. Summary of CD103^+^ proportion in iv) CD8^+^SIY pentamer^+^ CD103^+^ T cells, v) CD8^+^SIY pentamer^-^ CD103^+^ T cells. Each symbol represents one animal. Key: NS = not significant, *p < 0.05; **p < 0.01; ***p < 0.001; ****p < 0.0001.

To understand the mechanism by which pre-existing immunity affects the response to subsequent radiation therapy, we evaluated whether pre-existing immunity impacts the inflammatory environment of the tumor. Panc02-SIY tumors were established in mice left untreated or treated with anti-CD40L, and also in SIY tolerant mice and at d10 and d19, T cell responses to SIY were assessed and tumors were analyzed by multiplex cytokine assay. As shown previously, at early time points mice implanted with tumors had an SIY-specific T cell response detectable in the peripheral blood that was not found in anti-CD40L treated or SIY tolerant mice (Fig. 4ai). Surprisingly, the tumor inflammatory environment was not significantly different at early or late points between control, anti-CD40L treated and tolerant mice, despite the difference in the tumor antigen-specific T cell response. Multiplex analysis demonstrated no changes in a broad panel of cytokines and chemokines, with TNFα and IFNγ shown as examples of relevant inflammatory cytokines (Fig. 4aii-v). To determine whether these tumors differed significantly in T cell infiltrate, tumor infiltrating cells were isolated from day 14 tumors in mice treated with anti-CD40L or left untreated, and the immune infiltrate evaluated by flow cytometry. As previously shown, mice exhibited a strong antigen-specific response to SIY at day 7 which was blocked by anti-CD40L treatment, but declined by day 14 and tumor sizes were unchanged (Fig. 4b). The total tumor CD3^+^ T cell infiltrate was unchanged (not shown); however, there was a significant decrease in the number of CD3^+^CD8^+^ T cells but not CD3^+^CD4^+^ T cells per tumor (Fig. 4b). Surprisingly, the overall number of CD3^+^CD8^+^SIY-pentamer^+^ T cells per tumor was not significantly changed at day 14. To evaluate the function of these antigen-specific T cells in the tumor we made use of mice expressing the Nur77-GFP transgene, which is activated on TCR engagement^20,21^. Approximately a third of the CD3^+^CD8^+^SIY-pentamer^+^ T cells in the tumor had functionally engaged antigen in the past 24 hours, resulting in upregulation of Nur77-GFP (Fig. 4c). Consistent with the prior data, the overall proportion of CD8 T cells in the tumor was decreased in mice treated with anti-CD40L, but the proportion of SIY antigen-specific T cells remained approximately the same and among these cells the proportion recognizing antigen in the tumor was approximately the same (Fig. 4civ). Since antigen-specific T cells are found within the tumor but not in peripheral circulation, we examined their expression of resident memory markers. Almost all CD8^+^ T cells in the tumor are antigen experienced CD44^+^CD62L^−^ (not shown), but within this population are a subpopulation that are Ly6C^−^CD103^+^, which is consistent with a resident memory phenotype (Fig. 4di-iii)^22^. CD103^+^ T cells are present in both the antigen-specific CD3^+^CD8^+^SIY-pentamer^+^ T cells and the CD3^+^CD8^+^SIY-pentamer^-^ T cells of unknown specificity. In tumors, CD39 expression can define an exhausted population of CD8 T cells^23^. We find that the antigen-specific CD3^+^CD8^+^SIY-pentamer^+^ T cells have a larger population of CD103^+^CD39^+^ resident T cells than the CD3^+^CD8^+^SIY-pentamer^−^ T cells (Fig. 4div-vi). Strikingly, the CD103^+^ subpopulation of antigen-specific T cells is absent in mice treated with anti-CD40L at tumor implantation (Fig. 4ei-iv), and this population is also significantly decreased in the other CD8 T cells in the tumor (Fig. 4ev). These data demonstrate that the immune response at tumor implantation results in a population of exhausted tumor-specific T cells with a resident memory phenotype, and the presence of these cells correlates with responsiveness to radiation therapy and checkpoint inhibitors.

**Figure 5:**
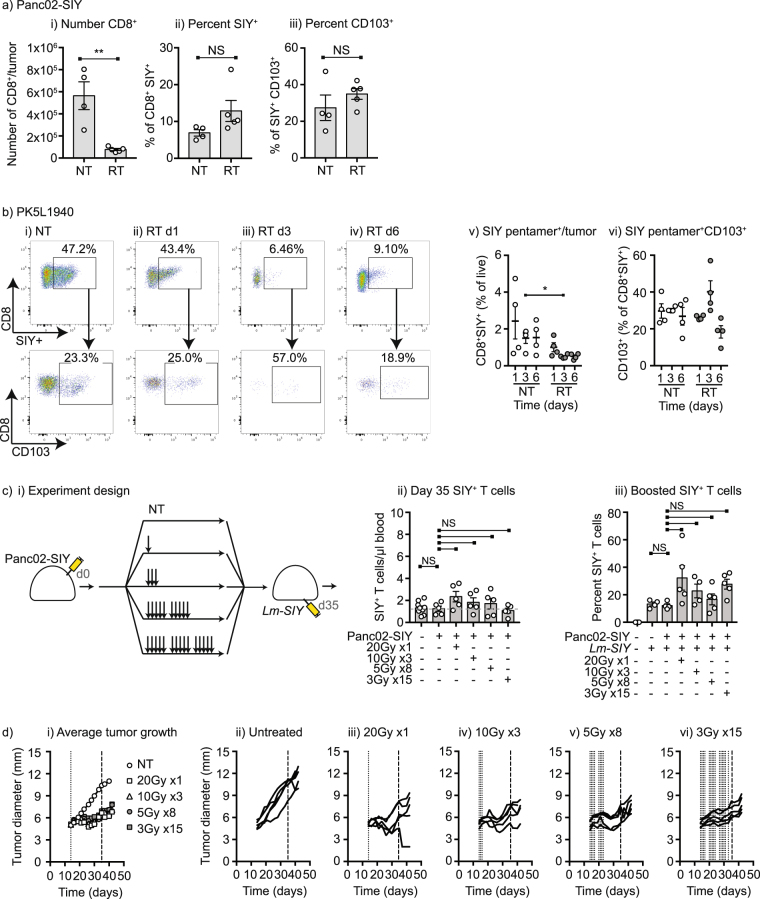
Radiation therapy to the tumor decreases but does not eliminate resident memory T cells. (**a**) i) Panc02-SIY tumors were implanted into C57BL/6 mice and left untreated (NT) or treated with 20 Gy focal RT on d14. Tumors were harvested 3 days later for analysis of tumor infiltrating i) CD8 T cells and ii) SIY-specific T cells. iii) CD103 expression in SIY-specific T cells. (**b**) PK5L1940 tumors were implanted into C57BL/6 mice and left untreated or treated with 20 Gy focal RT on d17. Tumors were harvested from i) untreated mice or ii) 1d, iii) 3d or iv) 6d following radiation therapy and analyzed for expression of CD103 in the SIY-specific tumor-infiltrating T cells. v) Summary of SIY-specific T cells per tumor. vi) Summary of CD103 expression in SIY-specific T cells. (**c**) i) Panc02-SIY tumors were implanted into C57BL/6 mice and left untreated (NT) or starting on d14 treated with a range of radiation schemes with equivalent BED. ii) At d35 following tumor challenge – at completion of all treatment schemes – mice were analyzed for SIY-specific T cells in the peripheral blood compared to naïve control mice. iii) Mice were subsequently challenged with Lm-SIY to boost antigen specific responses and analyzed 5 days later for SIY-specific T cells in the peripheral blood compared to naïve control mice. (**d**) i) Average tumor growth of mice treated as in c). Graphs show individual tumor responses in ii) untreated mice or mice treated with iii) 20 Gy x1; iv) 10 Gy x3; v) 5 Gy x8; or vi) 3 Gy x15. Key: *p < 0.05; **p < 0.01; ***p < 0.001; ****p < 0.0001; NS = not significant.

Since CD3^+^CD8^+^SIY-pentamer^+^ T cells are enriched in the tumor and are not peripherally circulating, these cells are entirely within the radiation field and are at risk of elimination following the local cytotoxic effects of radiation therapy. For this reason, we measured the effect of treatment on tumor-infiltrating CD8^+^SIY-pentamer^+^ T cells. Consistent with prior reports, the overall number of CD8^+^ T cells declined in the tumor 3 days following radiation, but the proportion of these surviving cells that were antigen-specific and the proportion of these that are CD103^+^ remained approximately the same (Fig. 5ai-iii). In tumors derived from spontaneous pancreatic tumors that express SIY, we similarly find that approximately one third of the CD8^+^SIY-pentamer^+^ T cells express CD103, and similarly while the proportion of CD8^+^SIY-pentamer^+^ T cells declines following radiation therapy, the proportion of these cells that are CD103^+^ fluctuates but does not significantly change following radiation therapy (Fig. 5b). To determine whether altering the dose of radiation therapy could permit peripheral recirculation of antigen-specific T cells, we tested a range of fractionated doses of radiation therapy that have a matched Biological Equivalent Dose (BED)^24^ (Fig. 5c). No radiation scheme restored peripheral antigen-specific T cell numbers and animals responded similarly poorly to antigen-specific boosting with systemic vaccination with *Lm-SIY* (Fig. 5d). These data are consistent with antigen specific T cells generated by tumor implantation being lost from peripheral circulation and establishing a resident memory niche in the tumor. Importantly, some proportion of the tumor-resident antigen specific T cells can survive high dose local radiation therapy, but do not return to peripheral recirculation in significant numbers.

**Figure 6:**
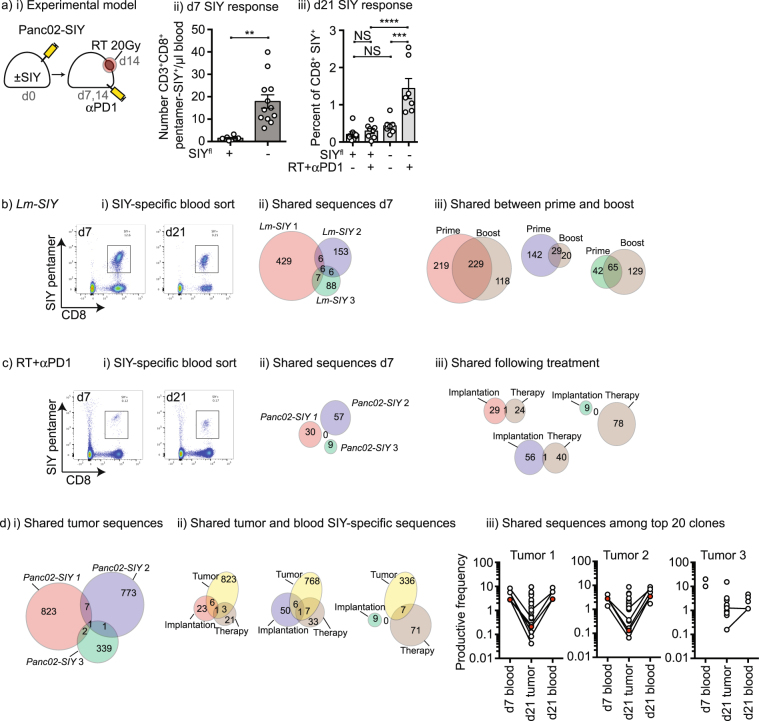
T cell responses generated by treatment are different from those generated by implantation. (**a**) i) Panc02-SIY tumors were implanted into wild-type mice or SIY^+^ mice and treated with anti-PD1 on days 7 and 14 and RT on d14. Blood was harvested from groups on ii) d7 and iii) d21 for analysis of SIY-specific T cells in the peripheral blood using SIY-pentamers. (**b**) i) C57BL/6 mice were vaccinated with Lm-SIY on d0 and d14, and peripheral blood was harvested on d7 and d21. SIY-specific CD8 T cells were sorted from the peripheral blood based on SIY-pentamer binding and sorted cells underwent TCRSeq to identify SIY-responsive clones. ii) The number of distinct SIY-specific clones in each animal at d7 following vaccine priming is shown, along with shared clones. Circle areas are approximately to scale. iii) The number of clones shared within individual animals between prime (d7) and boost (d21). Circle areas are approximately to scale. (**c**) i) Panc02-SIY tumors were implanted into C57BL/6 mice and treated with anti-PD1 on days 7 and 14 and RT on d14. Blood was harvested from mice on d7 and d21 and on d21 the tumor was also harvested. SIY-specific CD8 T cells were sorted from the peripheral blood based on SIY-pentamer binding and sorted cells underwent TCRSeq to identify SIY-responsive clones. ii) The number of distinct SIY-specific clones in each animal at d7 following vaccine priming is shown, along with shared clones. Circle areas are approximately to scale.0 iii) The number of clones shared within individual animals generated following implantation (d7) and treatment (d21). Circle areas are approximately to scale. (**d**) i)The number of distinct clones identified following TCRSeq of unfractionated tumors harvested at d21 is shown, along with shared clones. Circle areas are approximately to scale. ii) The number of clones shared between the tumor and those in the peripheral blood following implantation and following treatment. Circle areas are approximately to scale, but tumors are not shown to scale. iii) The top 20 most abundant clones in the peripheral blood at d7 and d21, and the top 20 most abundant clones in the tumor at d21 are shown. Lines linking symbols denote shared clones. Clones highlighted in red are found in all three samples. Key: NS = not significant, *p < 0.05; **p < 0.01; ***p < 0.001; ****p < 0.0001.

While radiation alone cannot release tumor-resident T cells, following combined radiation therapy and anti-PD1 we can again detect antigen-specific T cells in the peripheral blood (Fig. 6a). We hypothesized that in tumor-bearing mice, post-treatment T cells represent a re-expansion or re-distribution of prior T cell responses, rather than new T cells. To test this hypothesis we made use of TCR sequencing to follow individual antigen-specific T cell clones over time. To validate this approach, we vaccinated mice with *Lm*-SIY and sorted SIY-pentamer-specific T cells from the peripheral blood on d7; these mice were boosted with *Lm-*SIY on d14 and we sorted SIY-pentamer-specific T cells from the peripheral blood of the same mice on d21 (Fig. 6bi). Despite being genetically identical strains, TCR usage for antigen-specific cells varied between mice and there were few overlapping TCR sequences shared between animals (Fig. 6bii), requiring us to study the same animal before and after treatment. Following the boost vaccination with *Lm*-SIY, the most common TCR sequences remained dominant in the antigen-specific cells in the peripheral blood and overall approximately half of the TCR sequences were shared between primary and boost antigen-specific T cells (Fig. 6biii). These data demonstrate the feasibility of TCR sequencing antigen-specific T cells sorted from the peripheral blood and the necessity of testing the same animal over time. To test our hypothesis that post-treatment T cells represent a re-expansion of prior T cell responses, we purified SIY-specific pentamer binding CD8 T cells at day 7 following tumor implantation, and again at d21 following combined therapy (Fig. 6ci). Following implantation with Panc02-SIY, day 7 SIY-specific pentamer binding CD8 T cells shared no consistency in TCR sequence usage between mice (Fig. 6cii), so as with *L. monocytogenes* vaccinated mice, we followed the same mice before and after treatment. Unlike *L. monocytogenes* vaccination, few or no clones were shared between the antigen-specific T cells in mice before and after treatment, indicating that the T cells generated by treatment do not arise from the original T cell population generated by tumor implantation (Fig. 6ciii). These data mean that our hypothesis was false – the peripheral blood T cells generated by treatment are not the same cells detectable following tumor implantation. To determine whether the implantation or treatment clones are present in the treated tumors, we performed TCR sequencing on the tumors of these same animals on day 21. As anticipated, the tumors exhibited a much greater variation of TCR sequences (Fig. 6di) since these were not pre-selected for CD8^+^SIY-reactive cells. However, we could readily detect TCR clones in the tumor that were present in the SIY-specific blood T cells with no clear selectivity for those generated at implantation or treatment (Fig. 6dii). By comparing the relative abundance of sequences across sample sets, SIY-specific TCR clones found in the blood following implantation and following treatment are amongst the top 20 most abundant TCR clones in the tumor (Fig. 6diii). These data demonstrate that while clones generated at implantation disappear from the peripheral circulation they remain in the tumor, forming a tumor-resident T cell population that appears critical to treatment outcome. Nevertheless, treatment results in peripheral circulation of a novel population of T cells not detectable following implantation, which can also be found in the tumor. The relative importance of the tumor resident and circulating cells in control of the primary tumor and distant disease remain to be determined.

## Discussion

Our data demonstrates that in immunocompetent mouse models of transplantable tumors an anti-tumor T cell response is generated as an artifact of tumor implantation. This response wanes as tumors progress, and tumors also become unresponsive to T cell targeted checkpoint inhibitors over time. We demonstrate that radiation therapy, a means to cause large-scale release of antigenic material from tumors *in vivo*, can be used to restore tumor control by checkpoint inhibitors, but surprisingly we found that this effect is dependent on the T cells generated by tumor implantation. We demonstrate an approach to generate a silent tumor implantation that we propose can be used to screen for agents that generate new adaptive immune responses following radiation therapy.

Prior data from many investigators has clearly demonstrated that the combination of immunotherapy and radiation therapy causes regression of local, and in some cases distant tumors, mediated by CD8 T cells. However, the standard mechanistic model that we and others have used – depletion of CD8 T cells immediately prior to radiation – depletes both pre-existing tumor-specific CD8 T cells and those that would have been generated by the treatment. Thus, these experiments cannot separate the role of pre-existing immunity. While our experiments focus on the role of T cell responses to cancer and their impact on immune responses, other immune populations may participate in the control of tumors following radiation therapy. NK cells have been shown to provide some portion of tumor control following radiation therapy combinations^25^ and NK depletion can temporarily limit tumor control by radiation therapy and anti-PDL1 therapy^26^; however, the dominant mechanism of lasting tumor control in this model is via CD8 T cells. There have been examples of outstanding clinical responses to radiation therapy in patients receiving immunotherapy^27^, and evidence of distant tumor control in planned clinical trials^28,29^. Importantly, in both of these trials responders showed evidence of a different peripheral immune status to non-responders prior to the initiation of treatment^28,29^. In clinical trials of patients treated with the combination of anti-CTLA4 and radiation therapy outcomes have not been better than those anticipated with anti-CTLA4 alone^30^ and no randomized trials have yet reported. With currently available analyses of endogenous T cell responses, we cannot definitively prove that radiation therapy generated responses *de novo*, or acted as a boost vaccine for low-abundance pre-existing responses. Tumor-specific T cell precursor frequency has been shown to directly impact the ability of tumors to grow following implantation and can impact the quality of T cells generated following vaccination^31^. Multiple clinical studies have highlighted that patients with evidence of pre-existing immunity perform better on immunotherapy studies than those without^32,33^. In our murine model where strong defined antigens provide us an advantage in identifying anti-tumor immunity, antigen specific cell numbers rapidly decline below our detection limit in the circulation but are still required for successful therapy. We find that the dominant change in T cell responses influenced by pre-existing immunity can be found in the tumor environment, where T cells form a resident memory population that is associated with responsiveness to treatment. Our data demonstrates that tumors treated immediately following implantation are responsive to single agent immunotherapy. This is consistent with data in many models, and a very common intervention involves treating tumors within 3–5 days of implantation, often before a palpable mass can be detected. As the tumor progresses, the immune environment becomes increasingly complex and single agent immunotherapy generally becomes ineffective. By contrast, radiation therapy plus immunotherapy has shown efficacy on even on large, late stage tumors^34^ suggesting that this approach can revive pre-existing responses in situations much more relevant to the clinical scenario.

Recent studies have demonstrated a requirement for cross-priming to generate effective anti-tumor responses by the combination of immunotherapy and radiation therapy^35^. These models use Batf3 knockout mice that are deficient in the dendritic cell critical for cross-presentation of cell-associated antigens^5^. However, these also lacked the ability to cross-present cell associated antigen and generate immunity at tumor implantation. Batf3^+^ cross-presenting dendritic cells are critical for rapid rejection of highly immunogenic tumors following tumor implantation^5^ with the response dependent on STING activation in dendritic cells^36^, and early type I IFN production^36,37^, resulting in efficient cross presentation and CD8-mediated tumor clearance. Tumors injected into mice lacking STING or IFNAR1 that do progressively grow are also less responsive to subsequent tumor radiation therapy^6^, and in view of our data it is possible that this is due to a failure of immunity at implantation in addition to any potential effects on radiation-induced responses. Implantation of tumors as fragments rather than cell suspensions can result in immunological ignorance to the growing tumor, permitting highly immunogenic tumors to grow where the same cells implanted as suspensions are rejected^38,39^. Consistent with this, Zheng *et al*. demonstrated that where tumors were implanted as a fragment, antigen-specific vaccination was necessary for full control of tumors by radiation therapy plus checkpoint inhibition^40^. Interventions such as vaccination and local therapies such as radiation may be particularly relevant where the tumor exhibits lower levels of antigen that are insufficient to initiate *de novo* immune responses^41–43^. These data are important since vaccines targeting tumor-associated antigens have shown an ability to generate T cell responses, but have struggled to generate a clinical impact^44^. However, if these vaccines are considered as part of multimodality therapies including radiation therapy and immunotherapy, there is the potential for synergistic effects (reviewed in^45,46^).

Since we demonstrated that FTY720 treatment immediately prior to radiation therapy did not influence outcome, this suggests that antigen tracking to draining lymph nodes is not required for successful combination with checkpoint inhibitors. This is consistent with data in immunogenic melanoma models, where administration of FTY720 to mice with established tumors did not influence the outcome of anti-CTLA4 and anti-PD1 immunotherapy^47^. Importantly, in this model treatment with FTY720 at tumor implantation eliminated the efficacy of immunotherapy^47^, suggesting that as in our studies the T cells generated at tumor implantation are solely responsible for anti-tumor activity and support tumor-resident T cells as being critical for therapy. In addition our data agrees with observations reported by Dovedi *et al*., where in a model of radiation therapy (2 Gy x5) initiated 7 days following tumor challenge combined with anti-PD1, FTY720 delivered daily for up to 6 weeks beginning immediately prior to tumor implantation was more effective at blocking tumor cure than the same FTY720 treatment scheme beginning immediately prior to radiation^48^. However, in this approach, FTY720 treatment at implantation did not completely block responses and there remained a significant effect with FTY720 treatment at radiation therapy^48^. As we discussed, FTY720 does not completely block immune responses^13^, and it is possible that this lower fractionated dose given earlier in the growth of the tumor permits a greater opportunity for subsequent immune responses to initiate and develop than our larger doses to more established tumors. This is a critical area of research for the field in order to optimize the immune response to radiation therapy.

Despite these caveats, radiation therapy causes log fold death of cancer cells and this remains an opportunity to initiate new immune responses; however, our data shows that our current models are dominated by the effects of pre-existing immunity.

## Methods

### Animals and Cell Lines

6–8 week old C57BL/6 or BALB/c mice were obtained from Charles River Laboratories (Wilmington, MA) for use in these experiments. C57BL/6 Pdx-Cre^+/−^ mice were crossed with mice incorporating floxed Luciferase-SIY (Stock# 009043, The Jackson Laboratory) to generate Pdx-Cre^+/−^ SIY^+/−^ that express SIY in the pancreas. Pdx-Cre^+/−^ (Stock#014647, Jackson Laboratories, Bar Harbor, ME), Kras^(G12D)+/−^ (Stock#008179, Jackson Laboratories), and Trp53^(R172H)+/−^ (Stock#01XM2, NCI Fredrick Mouse Repository, subsequently back-crossed to C57BL/6 background) mice were crossed to generate Pdx-Cre^+/−^ Kras^(G12D)+/−^ Trp53^(R172H)+/−^ mice that generate pancreatic tumors^49^. These mice were further crossed to develop Pdx-Cre^+/−^ Kras^(G12D)+/−^ Trp53^(R172H)+/−^SIY^+/−^ mice that spontaneously develop pancreatic tumors expressing the model antigen SIY. Female prostate ovalbumin-expressing transgenic (POET) mice^50^ were kindly provided by Dr. Redmond (EACRI, Portland OR). Mice bearing the Nur77-GFP transgene were kindly provided by Dr. Moran (EACRI, Portland OR). Survival experiments were performed with 6–8 mice per experimental group, and mechanistic experiments with 4–6 mice per group. Animal protocols were approved by the Earle A. Chiles Research Institute IACUC (Animal Welfare Assurance No. A3913–01). All experiments were performed in accordance with relevant guidelines and regulations.

The Panc02 murine pancreatic adenocarcinoma cell line^51^ was kindly provided by Dr. Woo (Mount Sinai School of Medicine, NY). Panc02 cells expressing the model antigen SIY were kindly provided by Dr. Weishelbaum (University of Chicago, IL) as used previously^52^. The CT26 murine colorectal carcinoma^53^ was obtained from the ATCC (Manassas, VA). Established spontaneous pancreatic tumors from Pdx-Cre^+/−^ Kras^(G12D)+/−^ Trp53^(R172H)+/−^ or Pdx-Cre^+/−^ Kras^(G12D)+/−^ Trp53^(R172H)+/−^SIY^+^ mice were used to generate novel tumor cell line named PK51975B (SIY^-^) or PK5L1940 (SIY^+^). PK51975B cells were transfected with a plasmid expressing a GFP-SIINFEKL fusion protein or GFP alone to generate PK51975B-GFPova or PK51975B-GFP and sorted for cells with stable expression of GFP. Presentation of SIINFEKL was confirmed using a B3Z T cell assay^54^. Species identity checks on these murine cell lines were performed with murine-specific MHC antibodies, and were tested for contamination within the past 6 months using a Mycoplasma Detection Kit (SouthernBiotech, Birmingham, Alabama).

### Antibodies and Reagents

Fluorescently-conjugated antibodies CD11b-AF700, CD4-FITC, CD8-PerCP-Cy5.5, IFNγ-APC, CD3-e450, CD8-PerCP, CD4-FITC, CD4-e450, CD4-PerCP, and CD25-APC, were purchased from Ebioscience (San Diego, CA). CD8-PE-TxRD was purchased from Invitrogen (Carlsbad, CA). CD45-BUV395 and Fc block was purchased from BD Biosciences (San Jose, CA). CD90.2-AF700, CD103-AF647, Ly6C-BV711, CD8a- BV605, and Live/dead aqua were purchased from Biolegend (San Diego, CA).

Therapeutic anti-CTLA4 (clone 9D9), anti-CD40 (clone FGK4.5), and anti-CD40L (clone MR1) antibodies were obtained from BioXcell (Branford, CT) and resuspended in sterile PBS to a concentration of 1 mg/mL. Anti-PD1 (clone G1) was kindly provided by Dr. Andrew Weinberg (EACRI, Portland OR). Therapeutic checkpoint inhibitor antibodies were administered at 250 µg intraperitoneally. Depleting anti-CD8 antibody (YTS 169.4 – BioXCell, West Lebanon, NH) was given i.p. 50 µg two days before tumor implantation. DEC205ova was kindly provided by CellDex Therapeutics (Hampton, NJ) and administered together with 100 µg anti-CD40 both subcutaneously or both intravenously. FTY-720 was obtained from Cayman Chemical Company (Ann Arbor, MI) and administered as a single i.p. dose 24 hours prior to implantation. Anti-CD40L was delivered i.p. as 3 daily 250 µg doses to establish tumor implant tolerance.

PE-conjugated Kb- SIYRYYGL pentamers were purchased from Proimmune (Sarasota, FL). SIINFEKL-Kb tetramers were obtained from the NIH Tetramer Core Facility at Emory University (Atlanta, GA).

*ActA*-deleted (*ΔactA*) *Listeria monocytogenes* strains used for these studies were engineered to express the SIY peptide (*Lm-SIY*) or the SIINFEKL peptide of ovalbumin (*Lm-Ova*) cloned in frame with the ActA N-terminal fragment^55^. Bacteria were grown to stationary phase in brain-heart infusion broth, washed in PBS, and a dose of 1 × 10^7^ CFU was injected intravenously in 200 μL total volume and confirmed by plating of residual inoculum.

### Immunotherapy and Radiation Therapy of Tumors

Tumors were inoculated at a dose of 2 × 10^5^ Panc02, 5 × 10^6^ Panc02-SIY, 5 × 10^5^ PK51975B-GFPova, PK51975B-GFP, PK5L1940 (C57BL/6 mice), and 5 × 10^4^ CT26 (BALB/c mice). For combination therapy anti-CTLA4 was given at the previously determined optimal timing, which is 7 days prior to radiation therapy^10^. Immunotherapy with blocking antibodies to PD1 were given on day 7, 14 and 21, with radiation on d14^56^. CT-guided radiation therapy was delivered using a Small Animal Radiation Research Platform (SARRP, XStrahl, Gulmay Medical, Suwanee, GA) to an isocenter in the tumor, with beam angles designed to minimize dose to normal tissues. Dosimetry was performed using Murislice software (XStrahl). Alternative dose fractionations were selected to have a Biological Equivalent Dose matching 20 Gy x1 (Biological Equivalent Dose of 60 Gy). Tumor bearing mice were monitored a minimum of three days per week and euthanized when tumors exceeded 12 mm in any dimension, or when body condition score declined one level.

### Tumor-associated antigen specific responses

Quantitative assay of cell depletion or antigen-specific cell numbers in the peripheral blood were measured using a whole blood bead assay^57^. Briefly, whole blood was harvested into heparin tubes, and 50 µL was stained directly with fluorescent antibody cocktails along with PE-conjugated Kb- SIYRYYGL pentamers or Kb-SIINFEKL tetramers where appropriate. AccuCheck fluorescent beads (Invitrogen) were added to each sample, then red blood cells were lysed with BD FACS lysing solution (BD Biosciences), and samples analyzed on a BD LSRII flow cytometer. We determined the absolute number of cells in the sample based on comparing cellular events to bead events (cells/µL).

To measure T cell responses by intracellular cytokine stimulation (ICS), spleens were harvested 7 days following treatment and cell suspensions were stimulated with 2 µM of SIY peptide (SIYRYYGL), Ova peptide, (SIINFEKL), LLO peptide (GYKDGNEYI) or DMSO vehicle in the presence of brefeldin A for 4 hours at 37 °C. Stimulated cells were washed and stained with CD4-FITC and CD8-PerCP Cy5.5, then fixed and permeabilized using a BD Cytofix/Cytoperm plus kit (BD Biosciences) and frozen at −80 °C. For analysis cells were thawed and intracellularly stained with IFNγ-APC. Cells were washed and analyzed on a BD LSRII Flow Cytometer and the data was interrogated using BD FACSDiva (BD Biosciences) and FloJo (Tree Star, Ashland, OR).

### Tumor analysis

Detection of cytokines from tumor homogenates was performed using a murine multiplex bead assay as previously described^57^. Cytokine levels in the supernatants were detected using a murine multiplex bead assay (Life Technologies, Grand Island, NY) and read on a Luminex 100 array reader. Cytokine concentrations for replicates of each tumor sample were calculated according to a standard curve.

For flow cytometry of infiltrating cells, tumors were dissected into approximately 2 mm fragments followed by dissociation in a solution of 250 U/mL collagenase IV (Worthington Biochemical Corporation, Lakewood, NJ) and 30 U/mL DNase (Millipore Sigma, St Louis, MO) using a GentleMACS tissue dissociator (Miltenyi Biotech, Auburn, CA) and incubation at 37 °C for 30 minutes. The digest was quenched in serum and EDTA, then filtered through 100 µm nylon mesh to remove macroscopic debris and cell suspensions were washed and stained with antibodies as described previously^58^.

### TCRSeq

For isolation of T cells responding to vaccination, mice were vaccinated with 5 × 10^7^ CFU of *Lm-SIY* on d0 and d14 and peripheral blood from individual mice was harvested on d7 and d21. For isolation of T cells generated by tumor implantation and therapy, mice were implanted with Panc02-SIY on d0, treated with anti-PD1 on d7 and 14, 20 Gy focal radiation on d14, peripheral blood from individual mice was harvested on d7 and d21 and tumor was harvested on d21. In each case, CD8^+^SIY-pentamer^+^ T cells were sorted from peripheral blood. DNA was extracted from sorted T cells or unfractionated tumor using a DNAmicro kit (Qiagen), TCRSeq samples were prepared using an Adaptive immunoSEQ mouse T-cell Receptor kit (Adaptive Biotech, Seattle WA) and run on an Illumina HiSeq4000. Results were analyzed using an ImmunoSeq Analyzer (Adaptive Biotech).

### Statistics

Data were analyzed and graphed using Prism (GraphPad Software, La Jolla, CA). Individual data sets were compared using Student’s T-test and analysis across multiple groups was performed using ANOVA with individual groups assessed using Tukey’s comparison. Kaplan Meier survival curves were compared using a log-rank test.

### Data availability

All data generated or analyzed during this study are included in this published article (and its Supplementary Information files).

## Electronic supplementary material

Supplementary Figures
