# Supplementary material for: Tumor cure by radiation therapy and checkpoint inhibitors depends on pre-existing immunity
Source: Sci Rep. 2018 May 3;8:7012. doi: 10.1038/s41598-018-25482-w (PMC5934473; doi:10.1038/s41598-018-25482-w)
Supplement: Supplementary file 1 — Supplementary Figures [file 41598_2018_25482_MOESM1_ESM.pdf]

Tumor cure by radiation therapy and checkpoint inhibitors depends on pre-existing immunity

Marka R. Crittenden, Lauren Zebertavage, Gwen Kramer, Shelly Bambina, David Friedman, Victoria Troesch, Tiffany Blair, Jason R. Baird, Alejandro Alice, Michael J. Gough

Supplemental Figure 1

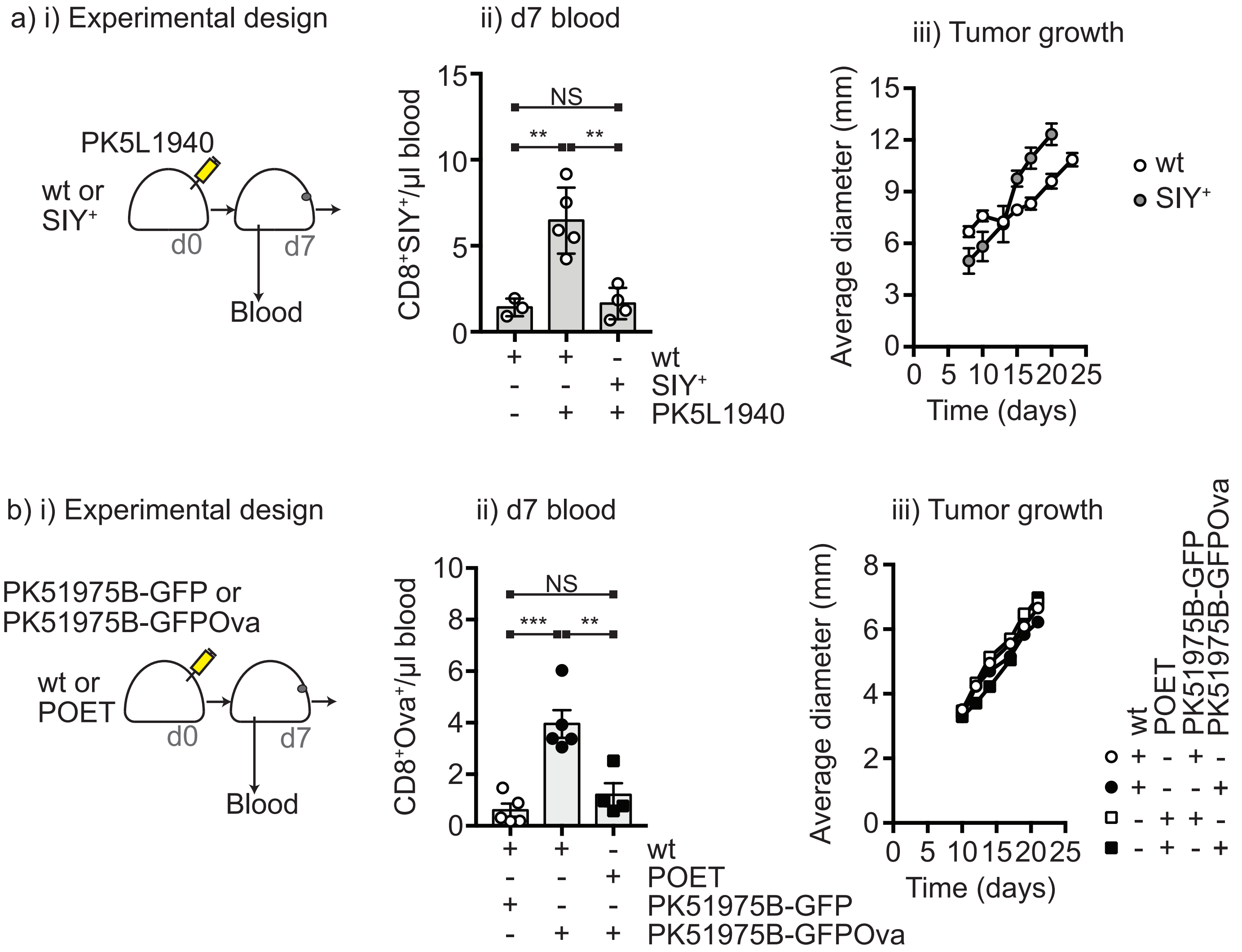

T cell response to tumor implantation in spontaneous pancreatic tumor models.
a) i) Wild type C57BL/6 mice or POET mice expressing ovalbumin in the prostate were implanted with 2x105 cells of a cell line derived from spontaneous pancreatic cancer in Pdx-Cre+/- Kras(G12D)+/- Trp53(R172H)+/- mice, engineered to express GFP (PK51975B-GFP) or a GFP-SIINFEKL fusion protein (PK51975B-GFPOva). ii) 7 days later peripheral blood was analyzed for the presence of CD8 T cells specific for Ova using SIINFEKL-tetramers. iii) Growth of PK51975B-GFP or PK51975B-GFPOva in Wild type C57BL/6 mice or POET mice. b) i) Wild type C57BL/6 mice or PDX-Cre+SIY+ mice expressing SIY in the pancreas were implanted with 2x105 cells from a cell line derived from spontaneous pancreatic cancer in Pdx-Cre+/- Kras(G12D)+/- Trp53(R172H)+/-SIY+ mice that endogenously expresses the SIY antigen (PK5L1940). ii) 7 days later peripheral blood was analyzed for the presence of CD8 T cells specific for SIY using SIY-pentamers. iii) Growth of PK5L1940 tumors in wild type C57BL/6 mice or PDX-Cre+SIY+ mice. Each symbol represents one animal. Key: NS = not significant, *=p<0.05; **=p<0.01; ***=p<0.001.

Tumor cure by radiation therapy and checkpoint inhibitors depends on pre-existing immunity

Marka R. Crittenden, Lauren Zebertavage, Gwen Kramer, Shelly Bambina, David Friedman, Victoria Troesch, Tiffany Blair, Jason R. Baird, Alejandro Alice, Michael J. Gough

Supplementary Figure 2

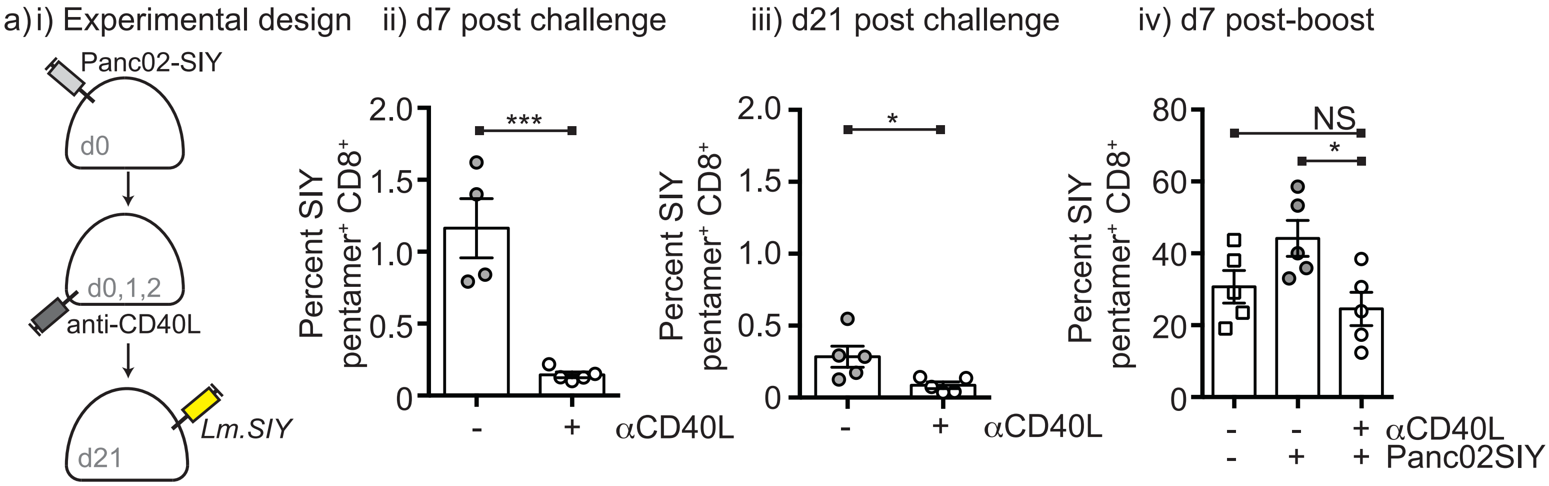

Anti-CD40L blocks the antigens-specific response to tumor implantation but does not eliminate antigen-specific cells.

a) i) C57BL/6 mice were implanted with Panc02-SIY tumors on d0 and left untreated or treated with 250μg anti-CD40L on d0, d1 and d2. Peripheral blood was analyzed for the presence of CD8 T cells specific for SIY using SIY-pentamers ii) 7 days or iii) 21 days following tumor challenge. iv) After analysis of blood on d21, tumor-bearing mice or a group of naïve mice were vaccinated by IV injection of Listeria monocytogenes expressing SIY (Lm.SIY) and analyzed for CD8 T cells specific for SIY in the spleen 7 days later. Each symbol represents one animal. Key: NS = not significant, *=p<0.05; **=p<0.01; ***=p<0.001.
